# Supplementary material for: Switchable Na+ and K+ selectivity in an amino acid functionalized 2D covalent organic framework membrane
Source: Nat Commun. 2022 Dec 22;13:7894. doi: 10.1038/s41467-022-35594-7 (PMC9780323; doi:10.1038/s41467-022-35594-7)
Supplement: Supplementary file 1 — Supplementary Information [file 41467_2022_35594_MOESM1_ESM.pdf]

Supplementary Information for

Switchable Na^+ and K^+ Selectivity in an Amino Acid Functionalized 2D Covalent Organic Framework Membrane

Li Cao [1], I-Chun Chen [1], Zhen Li [1], Xiaowei Liu [1], Muhammad Mubashir [1], Reham Al Nuaimi [1], Zhiping Lai [1*]

[1] Division of Physical Science and Engineering, 4700 King Abdullah University of Science and Technology (KAUST), Thuwal 23955-6900, Kingdom of Saudi Arabia.

*Corresponding author: zhiping.lai@kaust.edu.sa (Z. Lai).

Inventory of Supplementary Information:

Supplementary Methods

Supplementary Figures 1-27

Supplementary References 1-8

Supplementary Methods

Fabrication of COF-V-x% membranes on PAN support

COF-V-30% membrane

The synthesis procedure of COF-V-30% membrane on PAN support was similar to COF-V-60% membrane, except that 0.011 mM of PDA-V and 0.026 mM of PDA-OMe were used for the organic solution.

COF-V-80% membrane

The synthesis procedure of COF-V-80% membrane on PAN support was similar to COF-V-60% membrane, except that 0.05 mM of PDA-V and 0.0075 mM of PDA-OMe were used for the organic solution.

Fabrication of COF-Cys-x% membranes on PAN support

The COF-V-x% membranes can undergo an efficient thiol-ene reaction with L-cysteine after irradiation at 365 nm for 30 min with an initiator (H1361, 2-Hydroxy-4'-(2-hydroxyethoxy)-2-methylpropiophenone) to give the corresponding COF-Cys-x% membranes.

COF-Cys-30% membrane

A piece of COF-V-30% membrane was immersed into a 50 mL aqueous solution containing 121 mg of L-cysteine and 112 mg of H1361 initiator. The reaction mixture was then transferred to an UV crosslinker (XL-1000 UV CROSSLINKER, SPECTRO LINKER) and irradiated for 30 min at room temperature. The resulting COF-Cys-30% membrane was washed with deionized water (5 × 50 mL) to remove the residual L-cysteine and initiator. Finally, the COF-Cys-30% membrane was dried at 50 ^{o}C under vacuum for 24h.

COF-Cys-80% membrane

A piece of COF-V-80% membrane was immersed into a 50 mL aqueous solution containing 121 mg of L-cysteine and 112 mg of H1361 initiator. The reaction mixture was then transferred to an UV crosslinker (XL-1000 UV CROSSLINKER, SPECTRO LINKER) and irradiated for 30 min at room temperature. The

resulting COF-Cys-80% membrane was washed with deionized water (5 × 50 mL) to remove the residual L-cysteine and initiator. Finally, the COF-Cys-80% membrane was dried at 50 °C under vacuum for 24h.

COF-V-60% powder

COF-V-60% powder was synthesized according to the reference with a minor modification[1]. Typically, 0.05 mM of TAPB, 0.045 mM of PDA-V and 0.03 mM of PDA-OMe was dissolved in 10 mL acetonitrile by sonication for 5 min, followed by adding 0.5 mL 12 M acetic acid solution. The reaction mixture was kept undisturbed at room temperature (20 °C ± 2 °C) for 72h. The precipitate was collected by centrifugation, washed three times with acetonitrile, THF and ethanol, respectively. Finally, the powder collected was dried at 50 °C under vacuum for 24h in a yield of ~90%.

COF-V-30% powder

0.05 mM of TAPB, 0.0225 mM of PDA-V and 0.0525 mM of PDA-OMe was dissolved in 10 mL acetonitrile by sonication for 5 min, followed by adding 0.5 mL 12 M acetic acid solution. The reaction mixture was kept undisturbed at room temperature (20 °C ± 2 °C) for 72h. The precipitate was collected by centrifugation, washed three times with acetonitrile, THF and ethanol, respectively. Finally, the powder collected was dried at 50 °C under vacuum for 24h in a yield of ~92%.

COF-V-80% powder

0.05 mM of TAPB, 0.06 mM of PDA-V and 0.015 mM of PDA-OMe was dissolved in 10 mL acetonitrile by sonication for 5 min, followed by adding 0.5 mL 12 M acetic acid solution. The reaction mixture was kept undisturbed at room temperature (20 °C ± 2 °C) for 72h. The precipitate was collected by centrifugation, washed three times with acetonitrile, THF and ethanol, respectively. Finally, the powder collected was dried at 50 °C under vacuum for 24h in a yield of ~87%.

Characterization of membranes

Fourier-transform infrared spectroscopy (FT-IR)

FT-IR spectra of monomers, COF membranes and L-cysteine were recorded on a Nicolet iS10 FT-IR spectrometer equipped with an attenuated total reflection (ATR) in the wavenumber range of 4000-500 cm^{-1}

Solid-state ^{13}C cross-polarization nuclear magnetic resonance (^{13}C NMR)

Free-standing COF membranes were obtained by removing the PAN support in DMF. The solid-state ^{13}C NMR spectra of the free-standing COF membranes were recorded on a Bruker 400M WB NMR spectrometer.

X-ray photoelectron spectroscopy (XPS)

The X-ray photoelectron spectroscopy (XPS) analyses were carried out on the Kratos Axis Ultra DLD spectrometer with a monochromatic Al Kα X-ray source (1486.6 eV).

Powder X-ray diffraction (PXRD)

PXRD pattern of COF-V-x% powder was collected on a Bruker D8 Twin diffractometer equipped with a Cu Kα radiation source (λ = 1.5406 Å) operating at 40-kV acceleration and 40 mA from 1.5 to 30 ^{o} with a step size of 0.02^{o}.

Grazing incidence wide-angle X-ray scattering (GIWAXS)

GIWAXS measurements were carried out on a Bruker D8 Discover system equipped with a multimode Eiger 2 R 500 K 2D detector. Membrane samples were cut into 1 × 1 cm^2 pieces and mounted onto a silicon zero background sample holder. The grazing incidence angle was set at 0.5 ^{o}. Then, GIWAXS data was collected at room temperature over the 2θ range of 1.5-16° with an increment of 0.01^{o} and a 0.5 s /step.

Field-emission scanning electron microscopy (FE-SEM)

The surface morphology of COF membranes was observed by SEM on a Nova Nano 430 scanning electron microscope at the voltage of 5 kV and current of 56 pA, respectively. The membrane samples were coated with a 4-nm-thick iridium layer (Quorum Q150T sputter coater) before SEM imaging.

High resolution transmission electron microscope (HR-TEM)

Low-dose high resolution transmission electron microscope (HR-TEM) imaging were carried out on a Cs-corrected FEI G2 cubed Titan 60-300 electron microscope equipped with a Gatan K2 Summit direct-detection electron-counting camera operated under 300 kV. The COF active layer was first obtained by removing the PAN support in DMF and washed with DMF (5 × 30 mL) to remove the residual PAN polymer adsorbed on the COF active layer. Then the COF active layer was transferred onto a Cu grid for HR-TEM imaging.

Nitrogen sorption

The nitrogen sorption isotherms at 77 K were collected by using Micromeritics ASAP 2420 surface area and pore size analyzer. The free-standing COF active layer samples was first prepared by removing PAN support in DMF, and washed with DMF for 5 times to fully remove the PAN adsorbed on COF surface. ~50 mg of the COF active layer samples were degassed at 120 ^{o}C for 24h under vacuum and then subjected to N_2 sorption measurement. Brunauer-Emmett-Teller (BET) surface areas were calculated from the linear region of the N_2 isotherm in the P/P_0 range from 0.04 to 0.2 and the pore size distribution was calculated by using the non-local density functional model (NLDFT) method.

Ion diffusion experiment

Ion diffusion test was performed using a homemade H-shaped diffusion cell. COF membrane samples were mounted between two chambers (O-rings were used to seal the membrane). The effective membrane area is 0.5 cm^2. For single ion diffusion test, the feed side contains 30 mL of 0.1 M salt solution (KCl or NaCl) and the permeate side contains 30 mL of deionized water. The pH of feed side and permeate side solutions

was adjusted using 0.1 M HCl, KOH or NaOH solutions. The ion conductivity of the permeate side solution was recorded using a conductivity meter (CON2700, Eutech, USA). For all diffusion measurement, two chambers were stirred to avoid concentration polarization. For binary ion diffusion test, the feed side contains 30 mL of mixture solution of 0.1 M NaCl and 0.1 M KCl and the permeate side was filled with 30 mL of deionized water. In binary ion diffusion measurement, the concentration of K^+ and Na^+ in permeate side was measured by ICP-OES (PerkinElmer Optima 8300). The ion diffusion rate (J) of ions can be calculated from the following equation:

$$J = \frac{C \times V}{A \times \Delta t} \tag{1}$$

Where C and V are the salt concentration (M/L) and volume of the permeate solution (L), respectively; A refers to the effective membrane area (m^2) and Δt is the testing time (h).

The diffusion coefficient of Na^+ and K^+ through COF membranes can be calculated from the following equation[2]:

$$J = \frac{D}{d} \times \Delta C \times \frac{A_{eff}}{A} \tag{2}$$

where *J* is the permeation rate. ΔC is the concentration gradient (0.1 M). D refers to the diffusion coefficient and d is the thickness of the whole membrane (the thickness of the PAN support and COF membrane are ~150 μm and ~50 nm, respectively). A is the testing membrane area (0.5 cm^2) and A_{eff} is the effective pore area (~0.05 cm^{-2}).

Regulation of membrane potential

A piece of COF-Cys-60% membrane was mounted between two chambers of an electrochemical cell. Chamber A was filled with 100mM of NaCl and 5 mM of KCl solutions, and chamber B was filled with 100mM of KCl and 5 mM of NaCl solutions. Two Ag/AgCl electrodes (5 mm × 20 mm × 0.2 mm) were used to measure the membrane potential. The membrane potential of COF-Cys-60% membrane was continuously recorded using a source meter (KEITHLEY, 2450 sourceMeter®). The pH values of the two

solutions were switched between 3.8 and 8.9. After each pH adjustment, the membrane potential was recorded for 300 s.

Simulation details

All the MD simulations in this work were performed in the NVT ensemble by Gromacs 4.5.7 package[3]. The periodic boundary condition was applied to all of the three directions. A time step of 1 fs was used. A temperature of 298 K was maintained using the Nosé-Hoover thermostat[4]. The OPLS all-atom force field was used to describe the COFs[5]. The heavy atoms in COFs remained fixed in all the simulations. The particle mesh Ewald (PME) summation was used to calculate the long-range electrostatic interaction[6], with a cutoff of 1.3 nm for the separation of the direct and reciprocal space summation. The cutoff distance for the van der Waals interaction was 1.3 nm, and the parameters of the Lennard-Jones potential for the cross interactions between non-bonded atoms were obtained from the venerable Lorentz-Berthelot combination rule[7].

In all MD simulations, the simulation systems consisted ~67600 atoms with dimensions of approximately 7.24 × 6.27 × 15.0 nm^3, which were constructed by a graphene wall, a 5-layer COF membrane, ~ 20000 TIP3P water molecules[8] and ions ((i) K^+/Cl^- 1000 mM; (ii) Na^+/Cl^- 1000 mM, and (iii) K^+/Cl^- 1000 mM and Na^+/Cl^- 1000 mM). Here, a graphene was introduced into the simulation box to eliminate problems caused by finite boundaries, so that the COF membrane is the only pathway for ions transport between the left side and the right side of COF membrane. Then 60 ns simulation was performed for each system. The first 30 ns was discarded in each simulation trajectory for thermodynamic equilibration, followed by a 30 ns of production run.

The concentration of K^+/Na^+ is 1 M NaCl/1 M KCl for MD simulation, which can be explained as follows: Firstly, a 5-layer COF membrane was used in the simulation, which contains 120 e charges (-COO^-). Therefore, there should be 120 Na^+/K^+ to keep the system electrically neutral. Secondly, the time scale of MD simulation trajectories is typically on the order of tens of nanoseconds. Thus, additional K^+/Na^+ should

be available in the simulation box to study the transport behavior. That means the number of K^+/Na^+ should be more than 120 in the simulation box. Considering the volume of the simulation box, the minimum concentration of K^+/Na^+ should be 0.67 M. Therefore, the feed solution was 1 M Na^+/1 M K^+ for the MD simulation.

Supplementary Figures

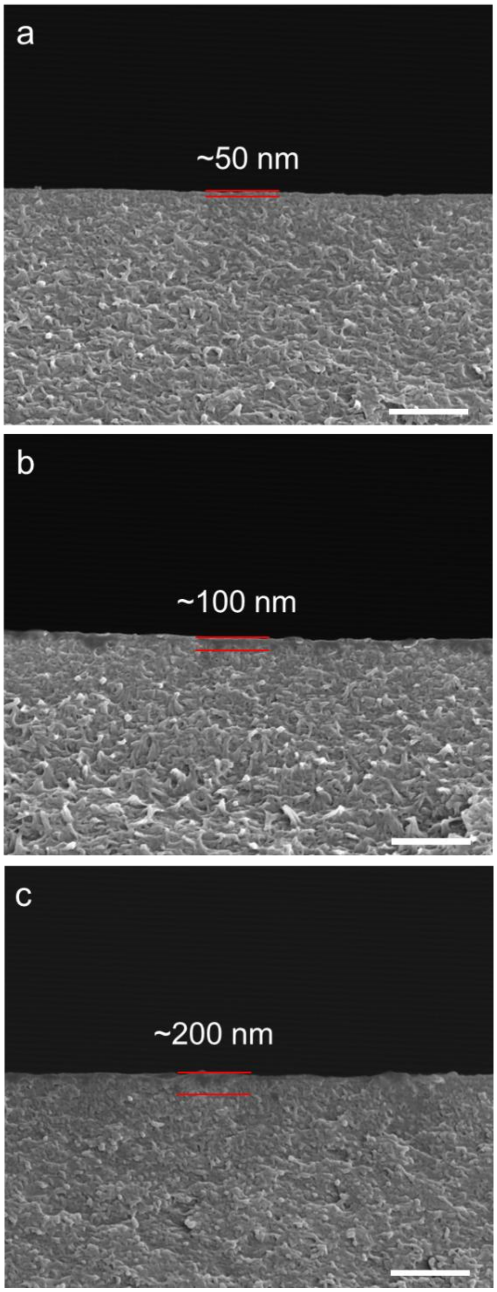

Supplementary Fig. 1 Cross-section SEM images of COF-V-60% membranes with various thickness. (a) Membrane thickness: 50 nm, scale bar, 500 nm; (b) membrane thickness: 100 nm, scale bar, 500 nm; (c) membrane thickness: 200 nm, scale bar, 500 nm

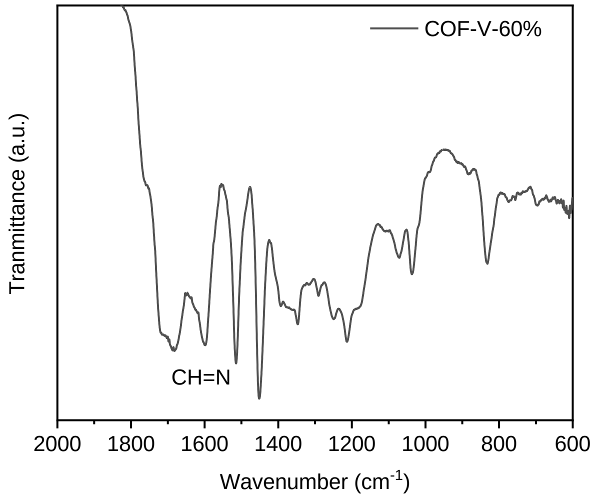

Supplementary Fig. 2 FT-IR spectrum of COF-V-60% membrane

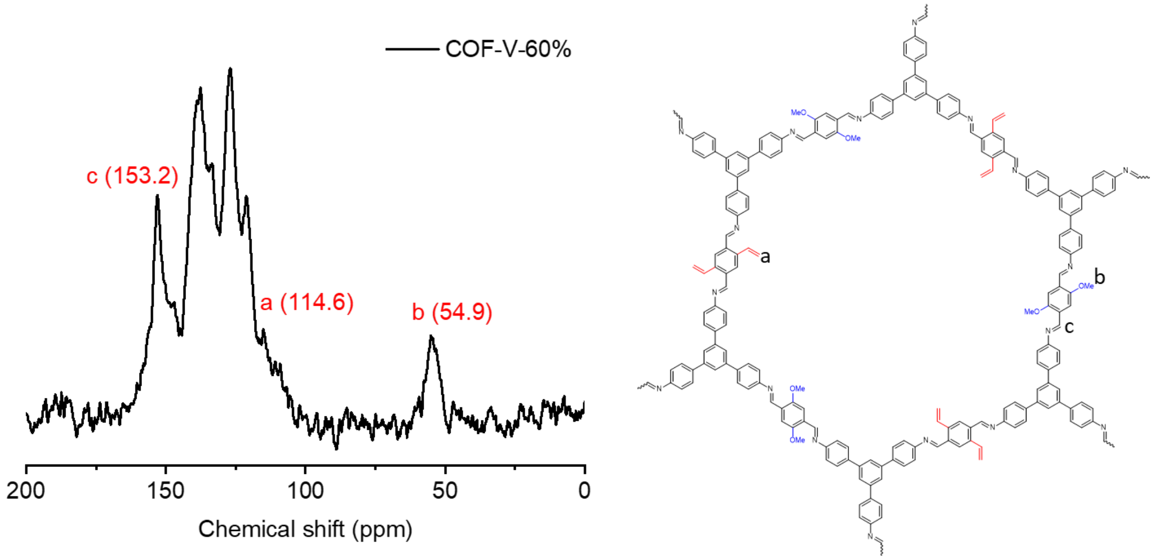

Supplementary Fig. 3 Solid-state ^{13}C nuclear magnetic resonance spectrum of COF-V-60% membrane.

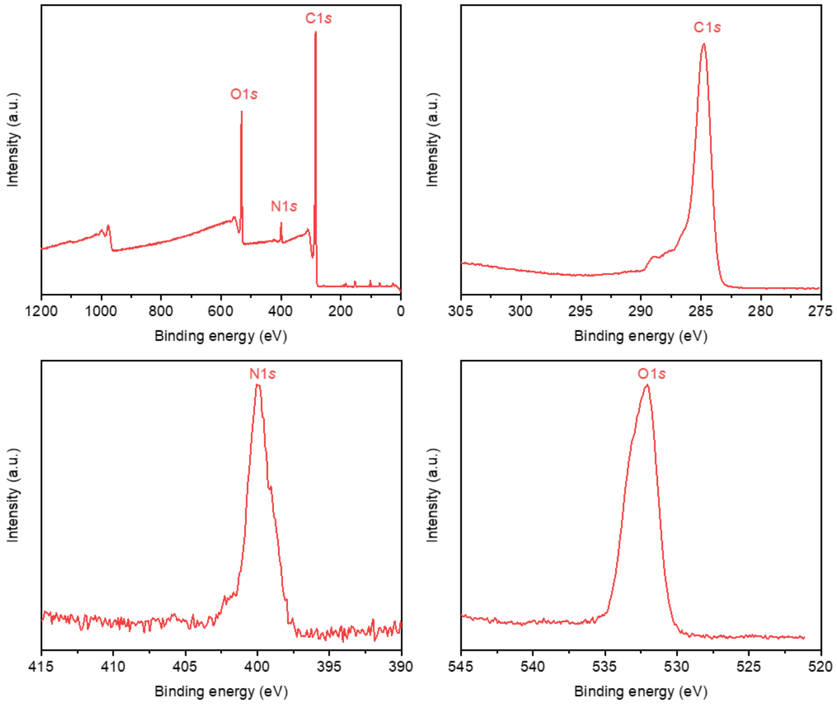

Supplementary Fig. 4 XPS spectra of COF-V-60% membrane

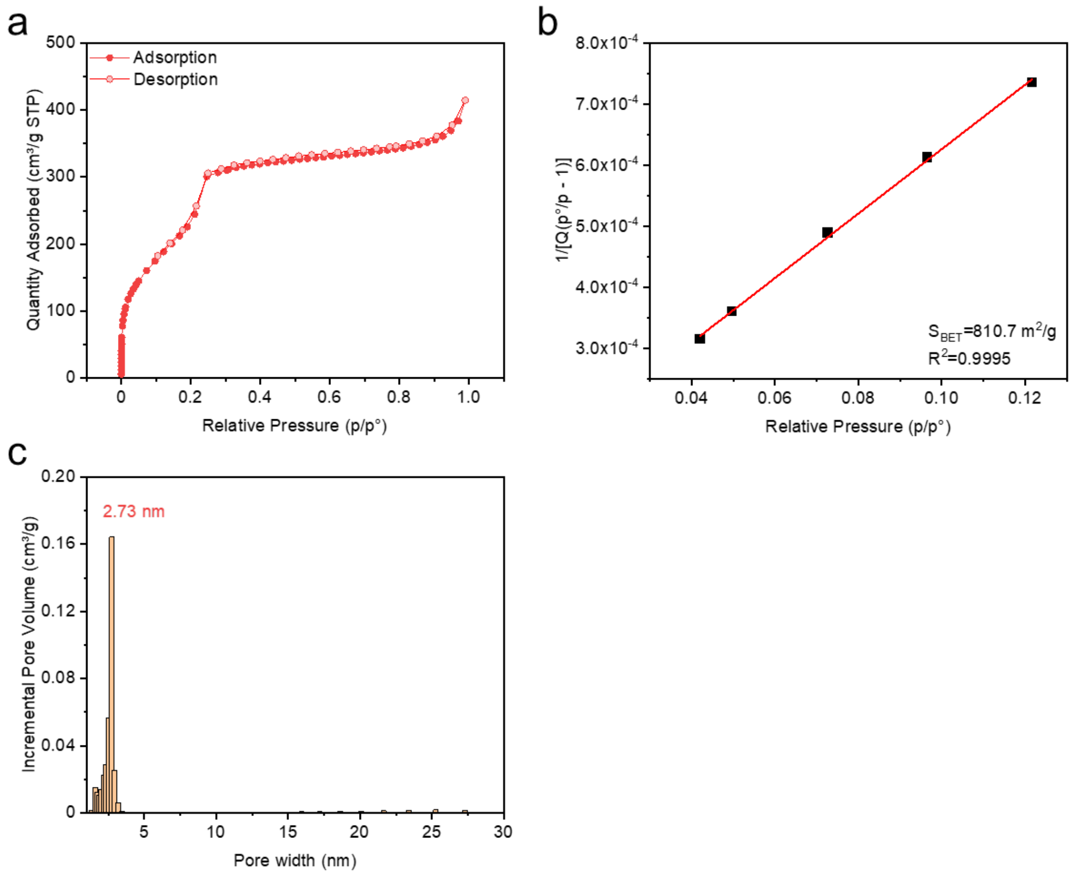

Supplementary Fig. 5 N_2 adsorption-desorption isotherms of COF-V-60% membranes measured at 77 K (a); BET plot (b); Pore size distribution profile calculated by using NLDFT model (c)

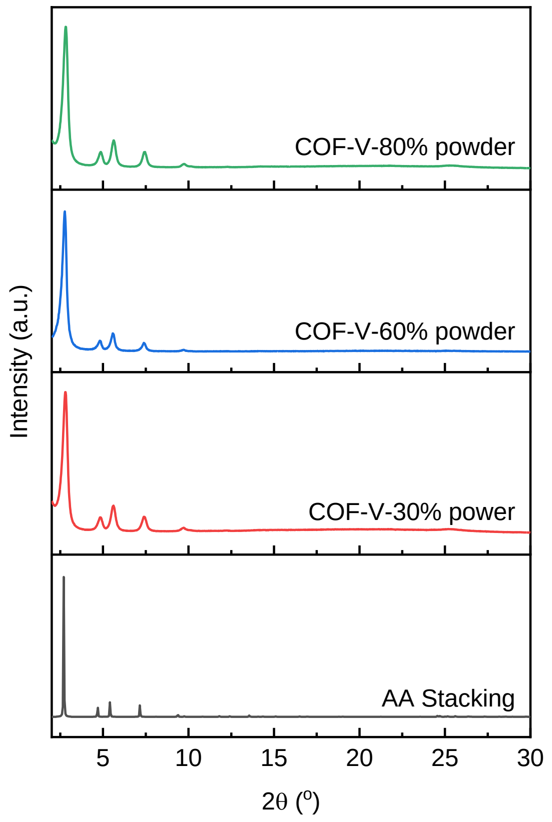

Supplementary Fig. 6 Powder XRD patterns of COF-V-x powder samples (x=30%, 60% and 80%).

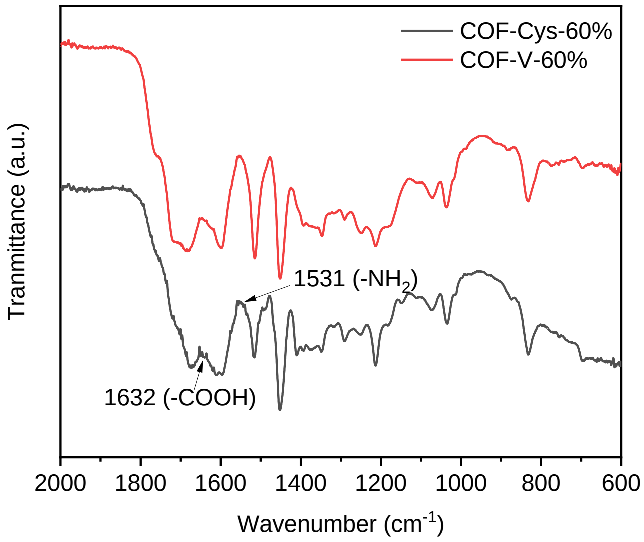

Supplementary Fig. 7 FT-IR spectra of the COF-V-60% membrane and COF-Cys-60% membrane.

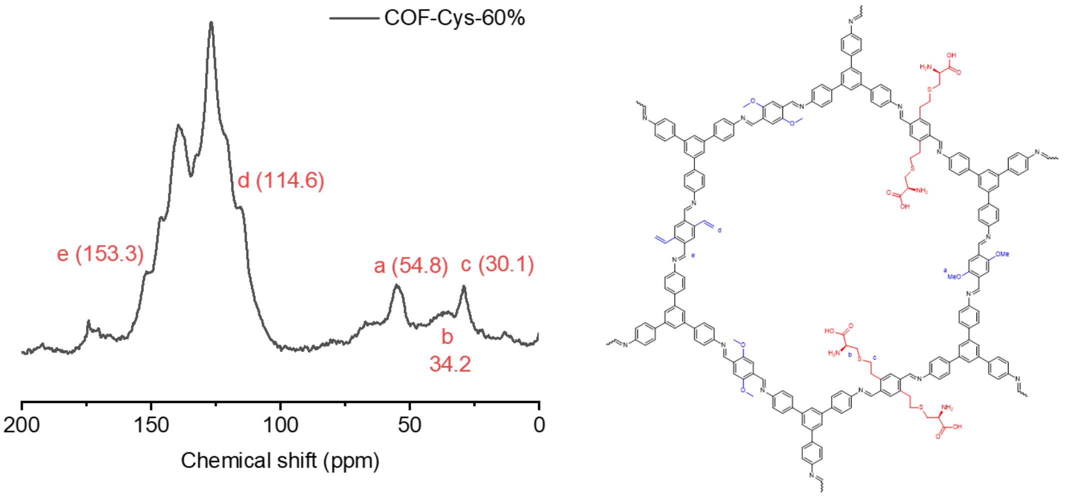

Supplementary Fig. 8 Solid-state ^{13}C nuclear magnetic resonance spectrum of COF-Cys-60% membrane.

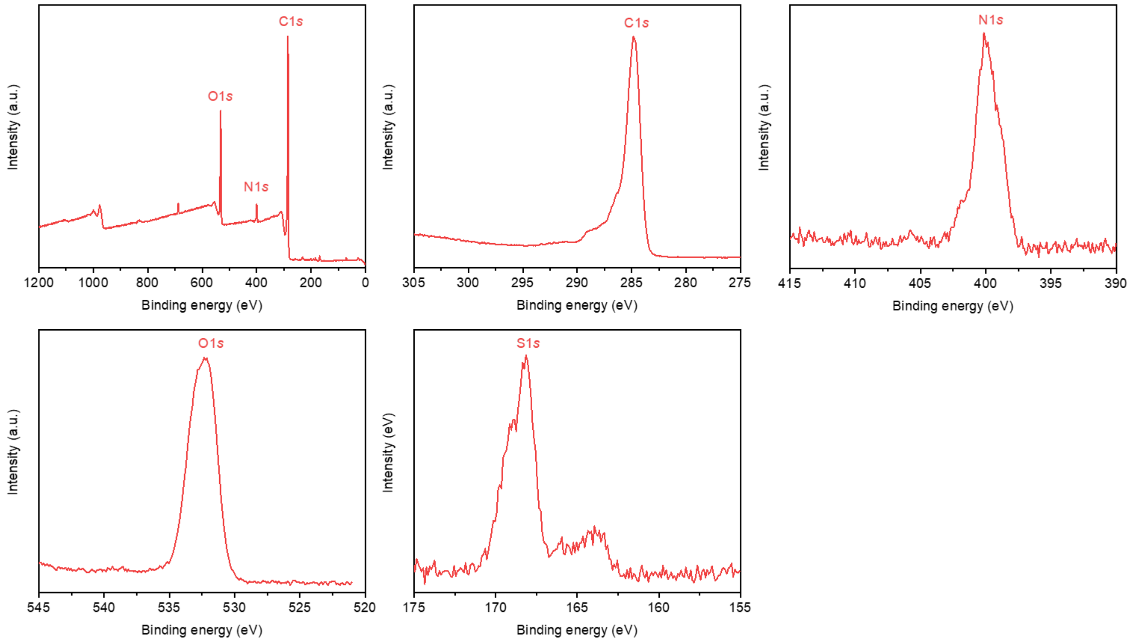

Supplementary Fig. 9 XPS spectra of COF-Cys-60% membrane

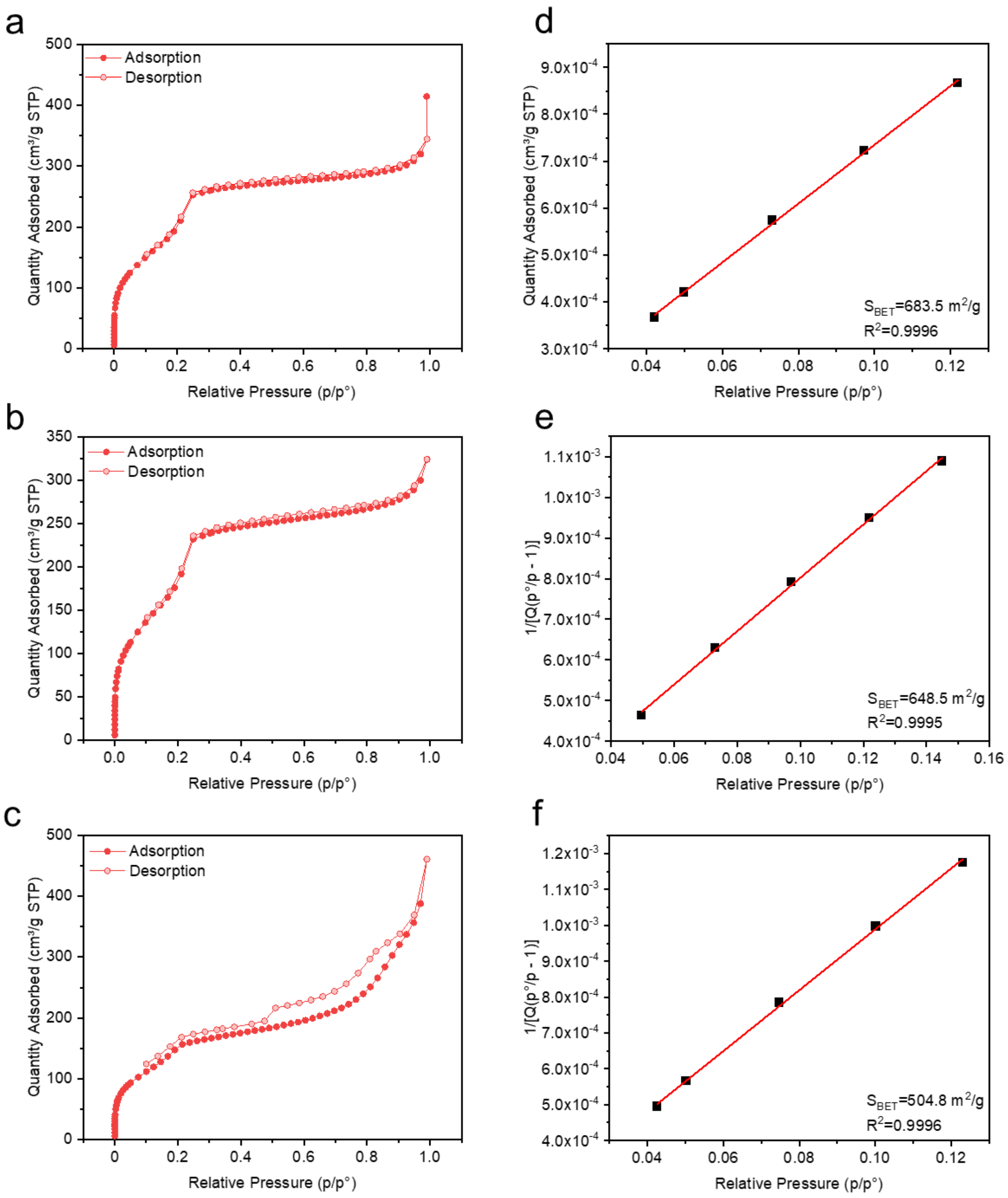

Supplementary Fig. 10 N_2 adsorption-desorption isotherms measured at 77 K for COF-Cys-30% membrane (a), COF-Cys-60% membrane (b), COF-Cys-80% membrane (c); BET plots for COF-Cys-30% membrane (d), COF-Cys-60% membrane (e), COF-Cys-80% membrane (f)

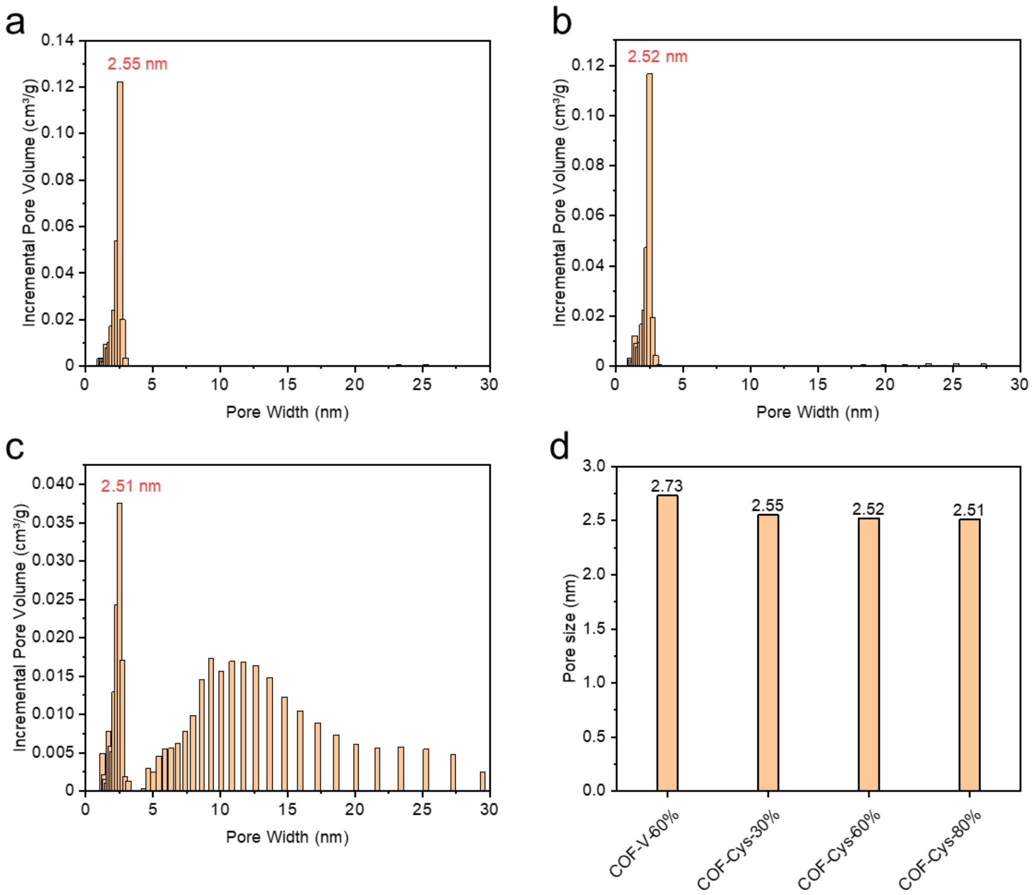

Supplementary Fig. 11 Pore size distribution profiles for COF-Cys-30% membrane (a), COF-Cys-60% membrane (b), COF-Cys-80% membrane (c), pore size as functions of various membranes (d)

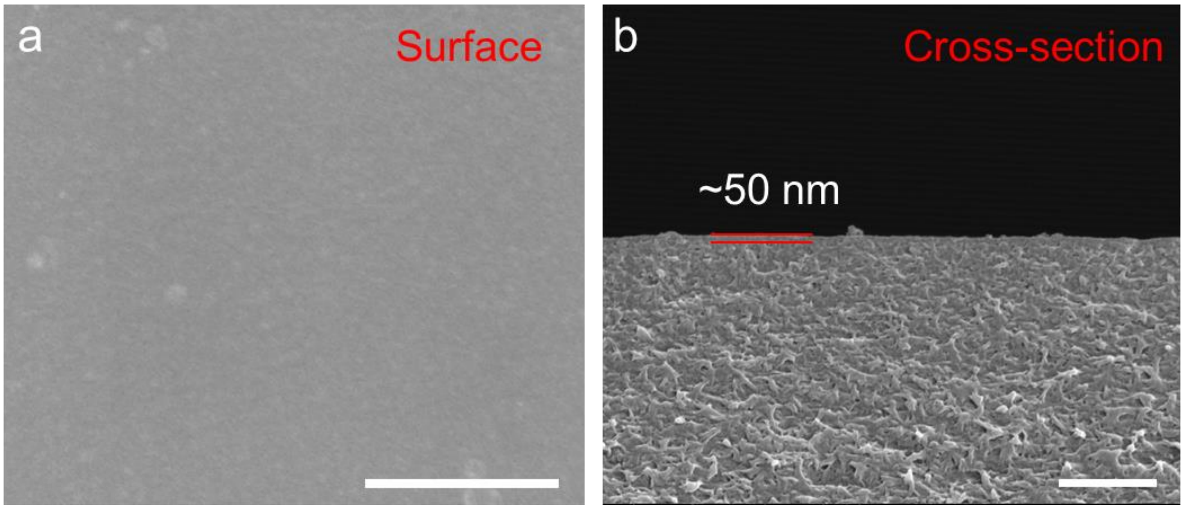

Supplementary Fig. 12 SEM images of COF-Cys-60% membranes: (a) Surface, (b) Cross-section. Scale bars in a and b, 500 nm.

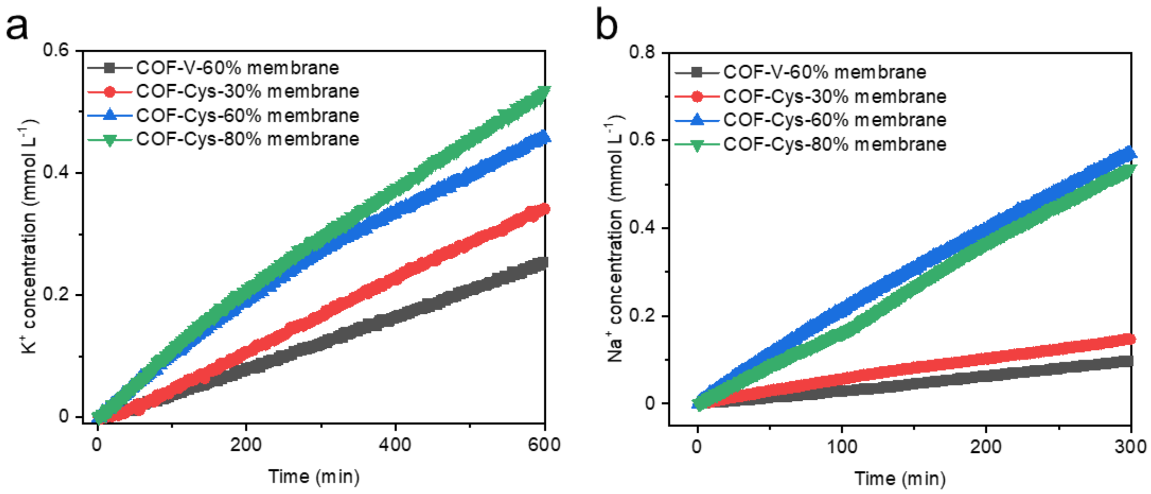

Supplementary Fig. 13 Ion concentration as functions of diffusion time measured in a single-ion system: (a) K^+ concentration in permeate side as a function of diffusion time; (b) Na^+ concentration in permeate side as a function of diffusion time

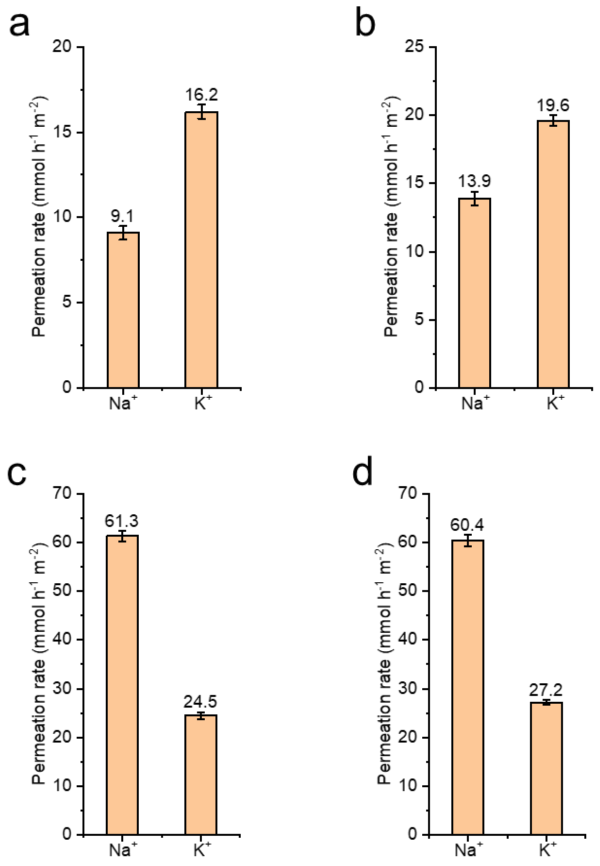

Supplementary Fig. 14 Permeation rate of COF-V-60% (a), COF-Cys-30% (b), COF-Cys-60% (c), COF-Cys-80% (d), tested in the single-ion system at pH 7.1. Error bars represent the standard deviation for three independent tests.

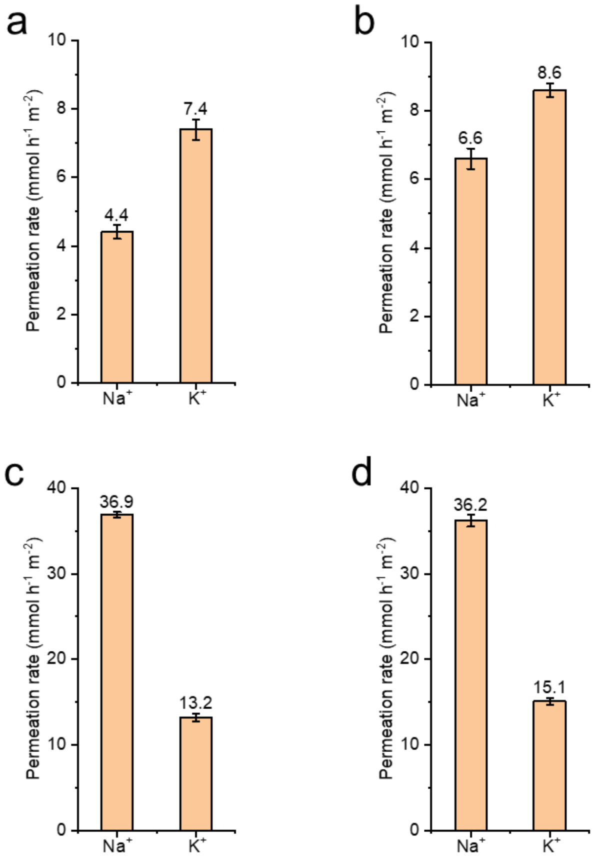

Supplementary Fig. 15 Permeation rate of COF-V-60% (a), COF-Cys-30% (b), COF-Cys-60% (c), COF-Cys-80% (d), tested in the binary-ion system at pH 7.1. Error bars represent the standard deviation for three independent tests.

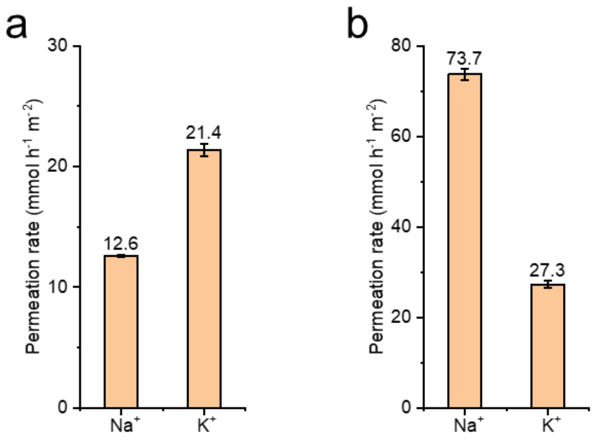

Supplementary Fig. 16 Permeation rate of Na^+ and K^+ for COF-Cys-60% membrane measured in a single-ion system at various pH conditions: (a) pH=3.8; (b) pH=8.9. Error bars represent the standard deviation for three independent tests.

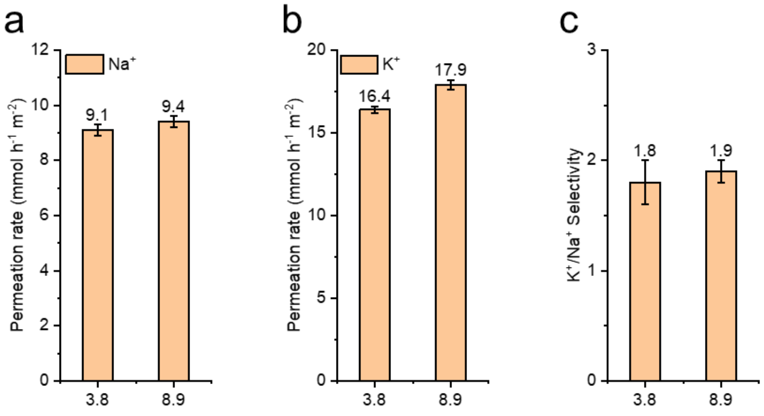

Supplementary Fig. 17 Permeation rate of Na^+ (a) and K^+ (b) for COF-V-60% membrane and corresponding K^+/Na^+ selectivity (c) at various pH conditions measured in the single-ion system. Error bars represent the standard deviation for three independent tests.

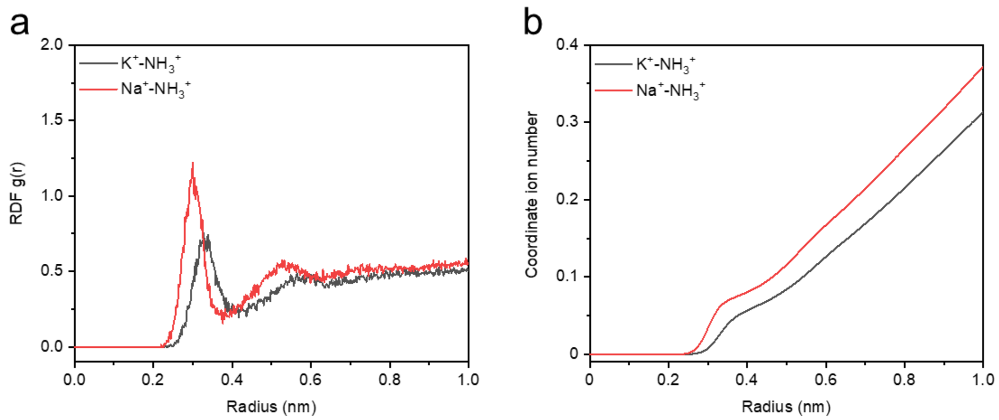

Supplementary Fig. 18 RDF of Na^+ and K^+ around NH_3^+ for COF-Cys-NH_3^+ membrane simulated in a 1M NaCl+1M KCl system (a); Coordinate ion number (b)

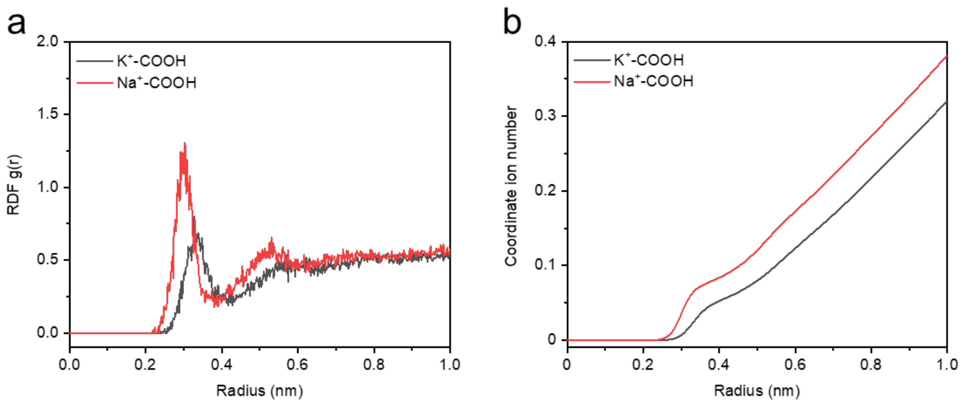

Supplementary Fig. 19 RDF of Na^+ and K^+ around COOH for COF-Cys-NH_3^+ membrane simulated in a 1M NaCl+1M KCl system (a); Coordinate ion number (b)

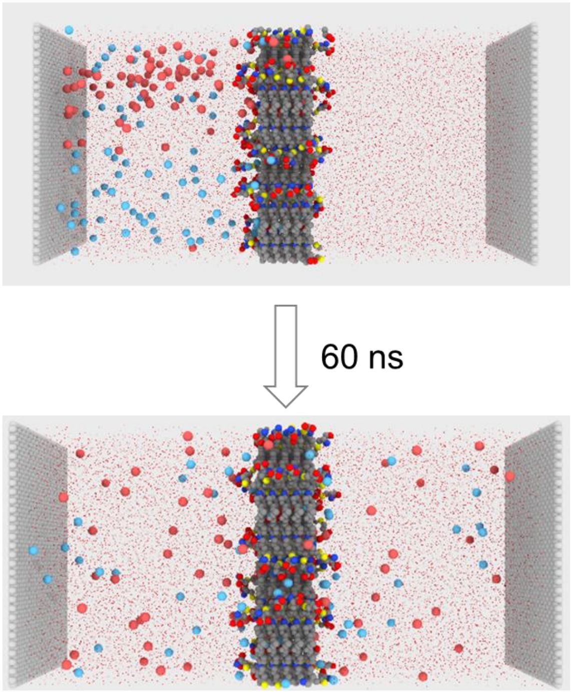

Supplementary Fig. 20 Molecular dynamics simulation.

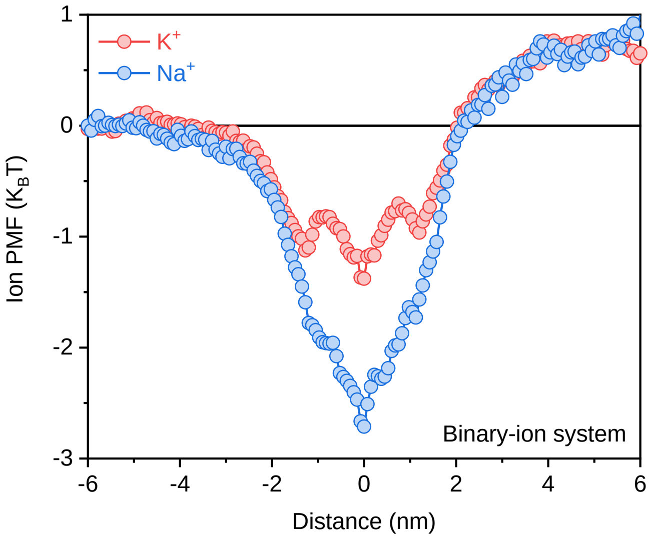

Supplementary Fig. 21 Ion PMF profile of COF-Cys-COO^- membrane

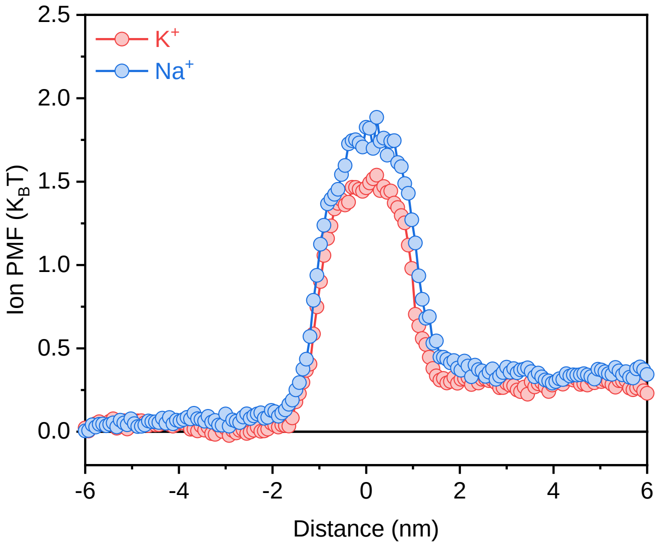

Supplementary Fig. 22 Ion PMF profile of COF-Cys-HN$_3^+$ membrane

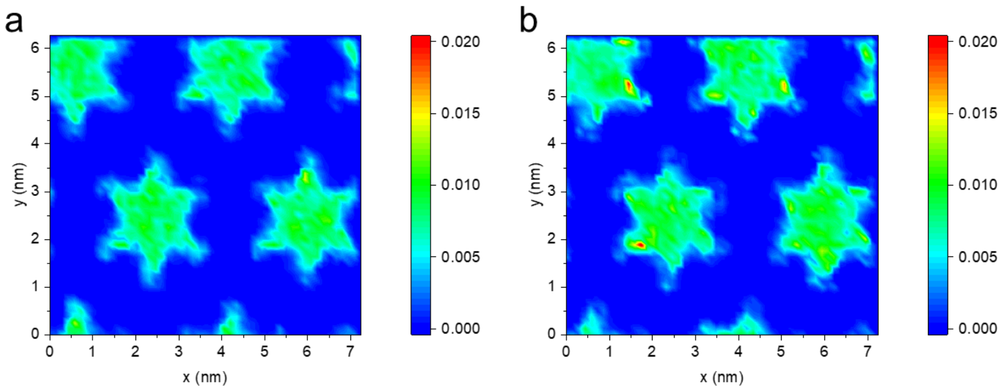

Supplementary Fig. 23 Density distribution profile of ions in xy-plane for COF-Cys-NH$_3^+$ membrane in the binary-ion system (1 M NaCl +1 M KCl): (a) Na$^+$; (b) K$^+$

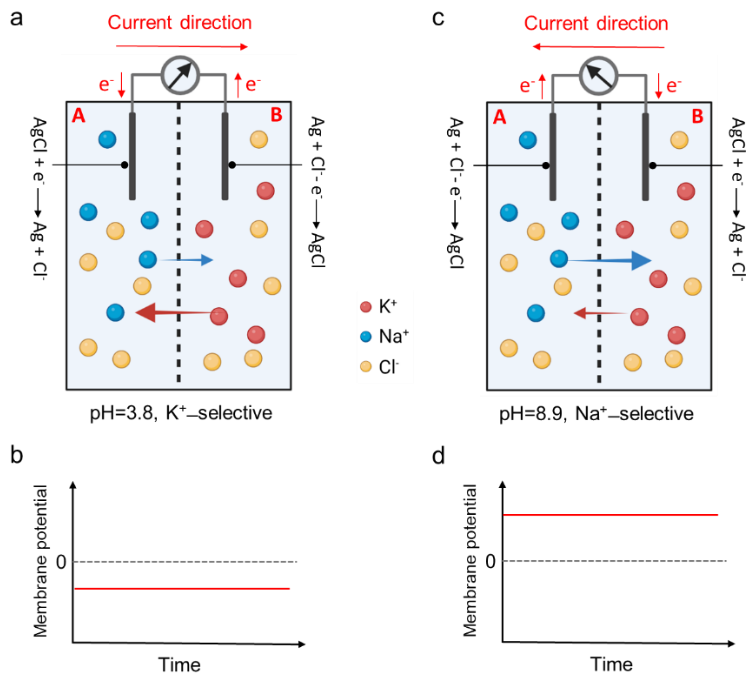

Supplementary Fig. 24 Demonstration of membrane potential under various pH conditions. Illustration of the generated current for the K^+-selective COF membrane (a) and the corresponding membrane potential (b); Illustration of the generated current for the Na^+-selective COF membrane (c) and the corresponding membrane potential (d).

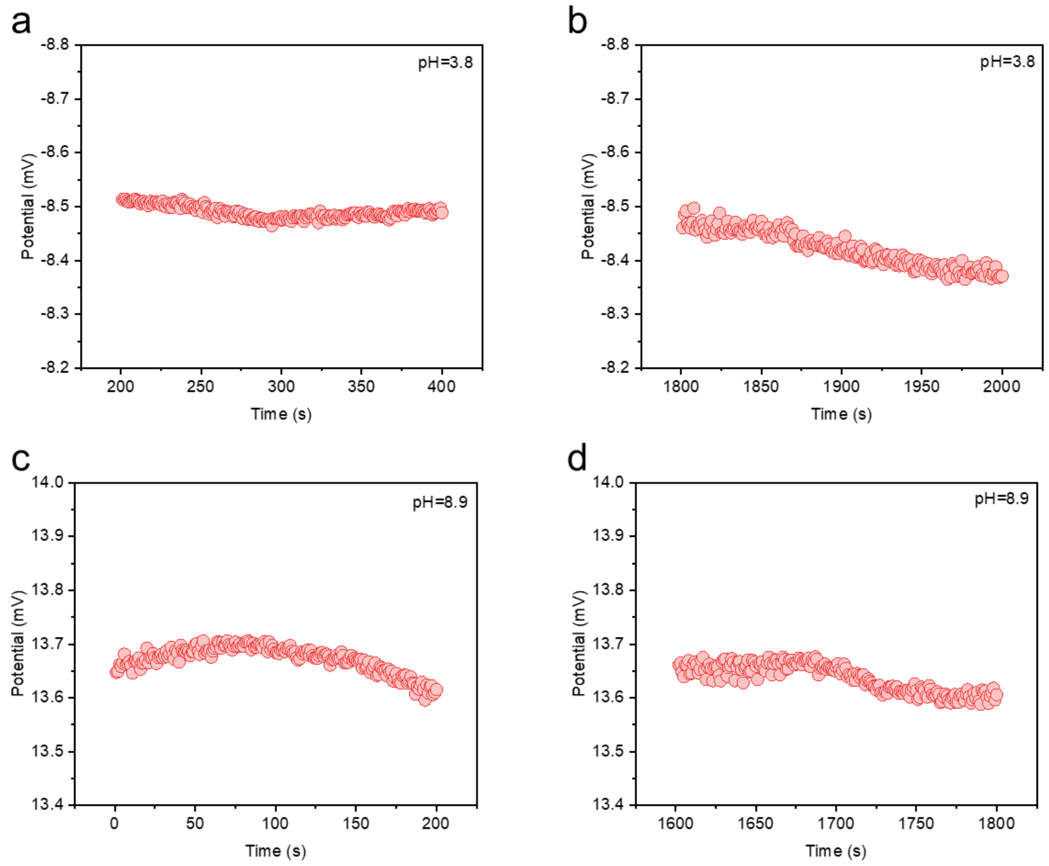

Supplementary Fig. 25 Membrane potential switches under different pH conditions. (a, b) Membrane potential at pH=3.8 condition; (c, d) membrane potential at pH=8.9 condition

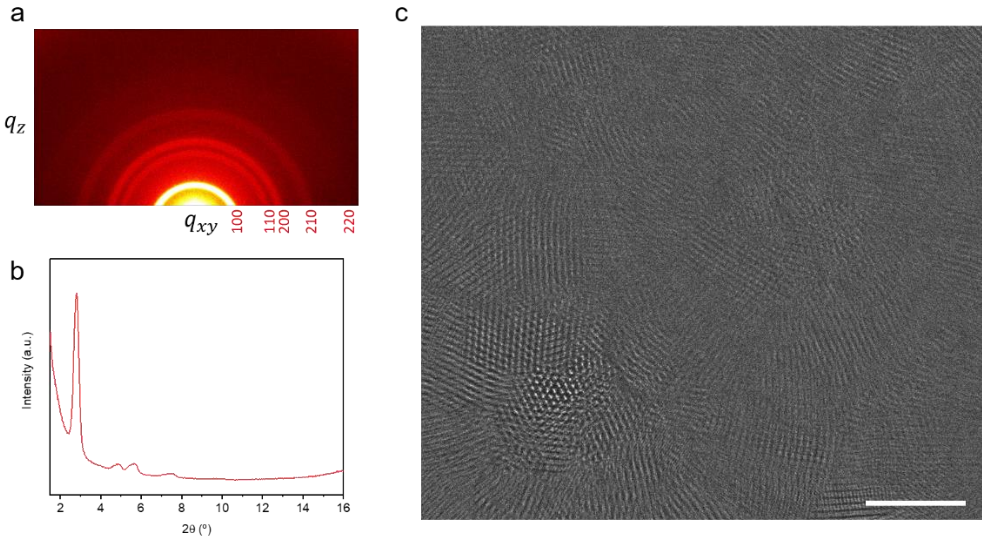

Supplementary Fig. 26 (a) GIWAXS data of COF-Cys-60% membrane after 5 cycles test. (b) Projection of the in-plane diffraction patterns from the GIWAXS data. (c) HR-TEM image of COF-Cys-60% membrane after 5 cycles test (scale bar, 50 nm)

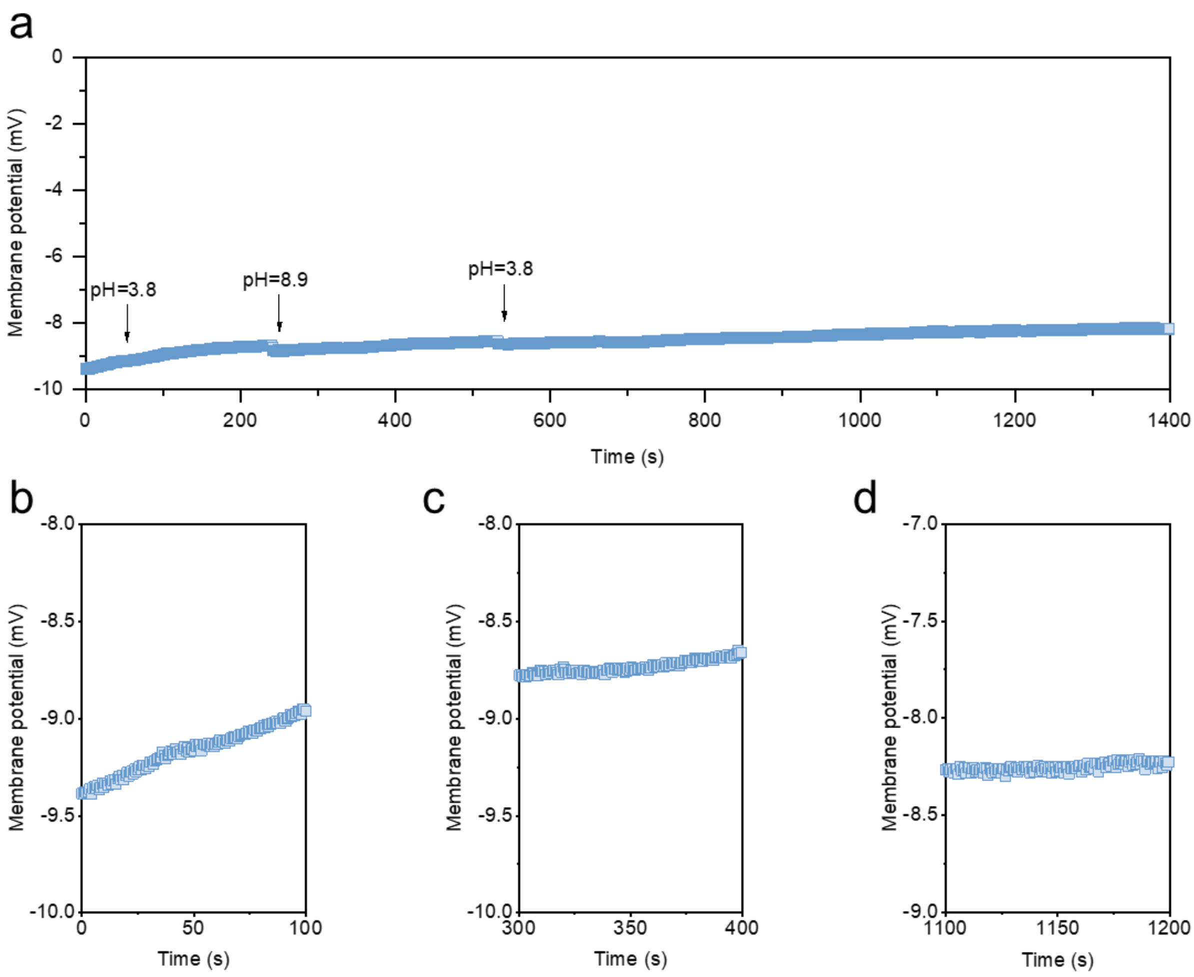

Supplementary Fig. 27 Membrane potential of COF-V-60% membrane at various pH conditions. (a) Membrane potential changes with the testing time. (b) Membrane potential at pH=3.8 condition; (c) Membrane potential at pH=8.9 condition; (d) Membrane potential at pH=3.8 condition (after pH=8.9).

Supplementary References:

1. Ma, W. *et al.* Size-Controllable Synthesis of Uniform Spherical Covalent Organic Frameworks at Room Temperature for Highly Efficient and Selective Enrichment of Hydrophobic Peptides. *J. Am. Chem. Soc.* **141**, 18271-18277 (2019).
2. Sheng, F. *et al.* Efficient Ion Sieving in Covalent Organic Framework Membranes with Sub-2-Nanometer Channels. *Adv. Mater.* **33**, 2104404 (2021).
3. Van Der Spoel, D. *et al.* GROMACS: fast, flexible, and free. *J. Comput. Chem.* **26**, 1701-1718 (2005).
4. Hoover, W. G. J. P. r. A. Canonical dynamics: Equilibrium phase-space distributions. *Phys. Rev. A* **31**, 1695 (1985).
5. MacKerell Jr, A. D. *et al.* All-atom empirical potential for molecular modeling and dynamics studies of proteins. *J. Phys. Chem. B* **102**, 3586-3616 (1998).
6. Darden, T., York, D. & Pedersen, L. J. T. J. o. c. p. Particle mesh Ewald: An N· log (N) method for Ewald sums in large systems. *J. Chem. Phys.* **98**, 10089-10092 (1993).
7. Hirsehfelder, J. O., Curtiss, C. F. & Bird, R. B. J. N. Y. Molecular theory of gases and liquids. *New York* (1954).
8. Jorgensen, W. L., Chandrasekhar, J., Madura, J. D., Impey, R. W. & Klein, M. L. J. T. J. o. c. p. Comparison of simple potential functions for simulating liquid water. *J. Chem. Phys.* **79**, 926-935 (1983).
